# Adaptable Hybrid Beamforming with Subset Optimization Algorithm for Multi-User Massive MIMO Systems

**DOI:** 10.3390/s24134189

**Published:** 2024-06-27

**Authors:** Ziyang Huang, Longcheng Yang, Weiqiang Tan, Han Wang

**Affiliations:** 1 School of Computer Science and Cyber Engineering, Guangzhou University, Guangzhou 510006, China; geekhzy@e.gzhu.edu.cn; 2 Sichuan Key Laboratory of Indoor Space Layout Optimization and Security Guarantee, Chengdu Normal University, Chengdu 611130, China; 151083@cdnu.edu.cn; 3 College of Physical Science and Technology, Yichun University, Yichun 336000, China; hanwang1214@126.com

**Keywords:** hybrid beamforming, intelligent reflecting surfaces, massive MIMO, channel models, regularized zero forcing, subset optimization

## Abstract

The exploiting of hybrid beamforming (HBF) in massive multiple-input multiple-output (MIMO) systems can enhance the system’s sum rate while reducing power consumption and hardware costs. However, designing an effective hybrid beamformer is challenging, and interference between multiple users can negatively impact system performance. In this paper, we develop a scheme called Subset Optimization Algorithm-Hybrid Beamforming (SOA-HBF) that is based on the subset optimization algorithm (SOA), which effectively reduces inter-user interference by dividing the users set into subsets while optimizing the hybrid beamformer to maximize system capacity. To validate the proposed scheme, we constructed a system model that incorporates an intelligent reflecting surface (IRS) to address obstacles between the base station (BS) and the users set, enabling efficient wireless communication. Simulation results indicate that the proposed scheme outperforms the baseline by approximately 8.1% to 59.1% under identical system settings. Furthermore, the proposed scheme was applied to a classical BS–users set link without obstacles; the results show its effectiveness in both mmWave massive MIMO and IRS-assisted fully connected hybrid beamforming systems.

## 1. Introduction

Convenient mobile communication in the Beyond 5th Generation (B5G) era provides a powerful impetus for the development of society, which also means that society has put forward higher requirements and expectations for mobile communication. With the access of wireless devices in the billions, mobile data traffic has produced an exponential explosion of rapid growth, and Ericsson’s official report shows that global mobile data traffic is expected to be as high as 136 EB (ExaBytes) per month in 2024 [1]. The advent of the B5G era demonstrates that mobile communication is no longer centered on the device alone, but rather on the user and the device at the same time. Unprecedentedly high transmission rates do not fully satisfy people’s needs, and richer spectrum resources, more diverse user experiences, and more flexible and free business requirements are the brand new challenges for the B5G era [2,3].

Massive multiple-input multiple-output (MIMO) allows tens to hundreds of antennas to be deployed at the base station (BS) end to serve a certain number of users, which can directly lead to significant sum-rate gains [4]. Massive MIMO technology also has two core advantages: One is that it can reduce inter-user interference through spatial diversity, and the other is that it reduces the system’s overhead by using very low transmit power compared to traditional MIMO techniques. Therefore, it is widely recognized in both academia and industry that massive MIMO is one of the key feasible technologies for B5G [5,6]. However, a problem that cannot be ignored is that a large number of antennas will undoubtedly bring high hardware cost as well as energy overhead.

As a traditional antenna technique, beamforming is an important part of our discussion of large-scale antenna arrays. The beamforming technique is a weighted combination of signals transmitted by multiple antennas with the user thereby forming the desired signals [7]. It can be categorized into analog beamforming (ABF) and digital beamforming (DBF), which have some differences in signal processing. Digital beamforming adjusts the signal phase and amplitude dynamically by digital means, the advantage is high flexibility and good signal adjustment, the disadvantage is that the energy overhead is large. Analog beamforming is generally through the phase shifter to adjust the signal phase, the advantage is that the realization has lower costs, the disadvantage is the flexibility of the signal adjustment effect is poor. To address this problem, Heath et al. proposed a technique called hybrid beamforming (HBF) [8]. The hybrid analog/digital architecture undoubtedly takes full advantage of both methods, reducing the number of expensive components such as RF chains and power amplifiers (PAs) far below the number of antennas, while also reducing the energy overhead of power-consuming devices such as analog to digital converters (ADCs) and digital to analog converters (DACs) as well as the processing of concurrent streams compared to the all-digital approach. This makes the HBF technique an effective solution to the high hardware cost and energy overhead of massive MIMO systems [9,10,11]. As a non-negligible signal processing process in the downlink transmission of the system, precoding utilizes the channel state information (CSI) at the transmitter to improve the system performance. Precoding has become a necessary functional component of the BS due to its strong interference suppression capability, which provides a considerable gains in the system’s sum rate [12]. Due to the difficulty of deployment and standardization, linear precoding is still more common than nonlinear precoding, although nonlinear precoding is generally superior to linear precoding. The two famous methods in linear precoding are zero forcing (ZF) and minimum mean square error (MMSE). The performance of these two methods varies in different channel conditions, and the appropriate method is usually chosen by weighing various factors. Regularized zero forcing (RZF) strikes a clever balance between the two methods by introducing a regularization term that takes into account both system’s capacity and interference suppression, which has attracted a lot of attention [13].

The current application environment of mobile communications is complex and varied. In previous studies, scholars have proposed numerous beamforming schemes for various channel environments: A. K. Hassan et al. analyzed the performance gains of beamforming performed in an MU-MIMO system under Rayleigh fading in the presence of co-channel interference [14]; S. Yan et al. proposed a location-based beamforming scheme (LBB) for Rician fading [15]; Y. Ding et al. proposed a low-complexity packet optimization algorithm for the mmWave channel [10]; Z. Lin et al. explored secrecy–energy efficient hybrid beamforming schemes for satellite–terrestrial networks [16]; and N. T. Nguyen et al. investigated two HBF schemes under cell-free (CF) mmWave massive MIMO systems [17].

As an emerging technology, the intelligent reflecting surface (IRS) is considered as a revolutionary technology for B5G due to its great advantages of good flexibility, programmability, low hardware cost, and low energy overhead while improving system performance. Hybrid beamforming assisted by IRS has also become a popular research direction: B. Di et al. designed an iterative HBF algorithm to perform digital beamforming and analog beamforming separately on BS and IRS [18]; L. Dai et al. proposed an inductive IRS structure to achieve CSI capture during beamforming [19] and Z. Lin et al. gave a joint beamforming optimization for IRS-aided hybrid satellite-terrestrial relay networks to minimize total transmit power while ensuring user rate requirements [20]. Although the traditional passive reflective surface is widely used and mature, its fixed signal reflection angle based on the fixed phase shifter in the work cannot be adapted to the complex and changing application environment of mobile communication. In recent years, the great success of RF microelectromechanical systems (MEMSs) and smart hypersurface materials have supported the vision of the automatic adjustment of signal reflection in mobile communication applications. The controller component of IRS automatically adjusts the passive programmable elements mounted on the reflective surface in real time in order to accomplish a variety of signal reflections with different amplitudes and phases. The reflected signals can be tuned to coherently phase with signals from other sources to improve the signal-to-noise ratio (SNR) at the target receiving end, or conversely, to perform destructive phase cancellation at the non-target receiving end to suppress interference, and thus, achieve the goal of securing the transmission in the IRS-assisted system [21,22].

Inspired by the aforementioned background and leveraging the methodologies described in [10,18], this paper proposes a subset optimization algorithm (SOA) for implementing adaptive hybrid beamforming in massive MIMO systems. Our motivation is to demonstrate the efficacy of the proposed scheme in enhancing communication quality while maintaining strong adaptability across different communication scenarios. To thoroughly validate the proposed scheme, we have implemented it in two distinct and common system models: a mmWave massive MIMO communication system without obstacles and an IRS-assisted massive MIMO system with obstacles. The former model evaluates the performance in a straightforward communication environment, while the latter assesses the scheme’s effectiveness in a more complex environment, where direct links are blocked and IRS is utilized to mitigate this issue. The specific contributions can be summarized as the following three points:

- Firstly, we propose a subset optimization algorithm-based hybrid beamforming (SOA-HBF) scheme for implementing adaptive HBF in massive MIMO systems. The SOA-HBF effectively reduces inter-user interference and improves system sum rate by dividing the users set into subsets based on inter-user correlation. Analog beamformers are pre-selected from the codebook for each subset, and the corresponding digital beamformer is computed using the RZF precoding algorithm. This method balances computational complexity and performance by sequentially removing selected vectors from the codebook, ensuring optimized analog precoding.
- Secondly, to demonstrate the effectiveness and adaptability of the proposed scheme, we evaluate it in a typical mmWave massive MIMO system without obstacles. This scenario assesses the performance in a straightforward communication environment, highlighting the proposed scheme’s capability. To further illustrate the adaptability of the scheme, we introduce an IRS-assisted massive MIMO system for scenarios with obstacles. The IRS is employed to mitigate the impact of obstacles and reconfigure the wireless propagation environment. Specifically, the IRS-assisted system is modeled using the traditional Rician channel to simulate the links between the BS, IRS, and the users set. This dual evaluation not only shows the performance of SOA-HBF in an ideal mmWave environment but also demonstrates the scheme’s capability to adapt to and enhance communication quality in more complex, obstacle-laden environments.
- Finally, extensive simulation experiments are conducted to validate the proposed scheme. By varying SNR and controlling other variables, we analyze the system’s performance across different scenarios. The results show that the SOA-HBF scheme significantly outperforms some existing HBF schemes, demonstrating strong adaptability and effectiveness in both obstacle-free and IRS-assisted communication environments. This confirms the scheme’s capability to enhance system’s capacity and reliability.

The rest of this paper is organized as follows. Section 2 outlines the hybrid beamforming system architecture and precoding algorithm employed in this paper. It introduces an IRS-assisted communication system with obstacles, while also discussing the problem formulation. Section 3 gives the concrete implementation of the proposed HBF scheme SOA-HBF based on the subset optimization algorithm SOA. Section 4 shows the results of the simulation experiments and the corresponding analysis. Section 5 draws conclusions and provides an outlook for future related work.

*Notation*: In the mathematical notation used in this article, bold uppercase represents matrices, bold lowercase represents vectors, and regular letters represent scalars. For a generic matrix S, $S^{H}$, $S^{T}$, $S^{-1}$, $S_{m,n}$ corresponds to the Hermitian transpose, transpose, inverse operation, and the element of the *m*th row and *n*th column, respectively. In addition, |·| denotes the absolute value, E(·) denotes the expectation, ∥·∥ denotes the l2-paradigm, Ω(·) denotes the number of elements in the set, and ⊗ denotes the Kronecker product. Finally, $I_{K}$ is a K×K unit matrix, 1 is an all-1 matrix, and 0 is an all-0 matrix.

## 2. System Model and Problem Formulation

In this section, we first establish a downlink system with fully connected hybrid beamforming architecture for narrowband communication of multiple users, and then, the channel model and precoding algorithm that will be used in the established system model are introduced, and finally, we provide the problem formulation based on the above analysis. In addition, considering the existence of inter-user interference and the system energy efficiency, we normalize the system transmit power, i.e., $P_{t}=1$.

### 2.1. System Model

**Figure 1:**
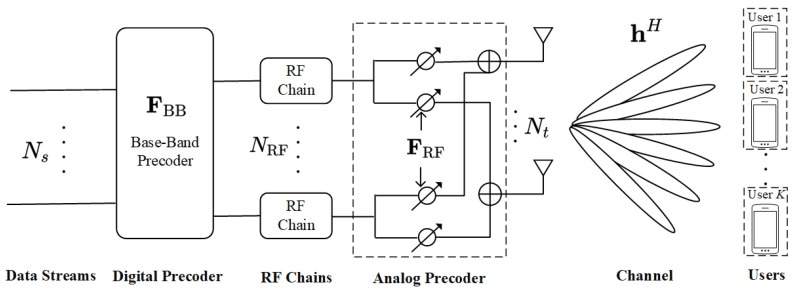
Fully connected hybrid architecture for multiple-users massive MIMO system.

We consider a multiple-users massive MIMO fully connected hybrid beamforming architecture for the downlink; since channel estimation is not the main focus of this paper, we assume perfect CSI for a given system. For the massive MIMO system as shown in Figure 1, it is assumed that the BS uses a uniform linear array (ULA) equipped with $N_{t}$ antennas, and the BS transmits the $N_{s}$ data streams by multiplexing to the users set at the receiving end, which has a total of *K* single-antenna users. For the hybrid beamforming matrix at the BS, we have $F=F_{\mathrm{RF}}F_{\mathrm{BB}}$, where the low-dimensional digital beamforming matrix is $F_{\mathrm{BB}}\in C^{N_{\mathrm{RF}}\times N_{s}}$, and the high-dimensional analog beamforming matrix is $F_{\mathrm{RF}}\in C^{N_{t}\times N_{\mathrm{RF}}}$. Combined with the feature that ABF is based on phase shifter implementation, the RF chains at the transmitter are connected to the transmitter antenna through phase shifters, and their number $N_{\mathrm{RF}}$ satisfies $N_{s}\leq N_{\mathrm{RF}}\ll N_{t}$. In addition, the subsequent appearance of ${\left(F_{\mathrm{RF}}F_{\mathrm{BB}}\right)}_{k}$ represents $F_{\mathrm{RF},k}$ multiplied by $F_{\mathrm{BB},k}$. Meanwhile, in this paper we set $N_{s}=N_{\mathrm{RF}}=K$. According to [23], the symbol vector $s\in C^{N_{s}\times 1}$ emitted by the BS satisfies the normalized emitted power $E\left\{{\mathrm{ss}}^{H}\right\}=\frac{1}{N_{s}}I_{N_{s}}$; the expression for the hybrid beamformer output signal vector $x\in C^{N_{t}\times 1}$ is

(1) $$x=F_{\mathrm{RF}}F_{\mathrm{BB}}s=\sum_{k}^{K}{\left(F_{\mathrm{RF}}F_{\mathrm{BB}}\right)}_{k}s_{k}.$$

The channel model $h\in C^{N_{t}\times 1}$ used in the system is tasked with conveying the signal x from the transmitter of the system to the receiver, and the signals received by the user *k*, (k=1,2,⋯,K) in the users set can be expressed as

(2) $$r_{k}=h_{k}^{H}x+n,$$

where $n\in C^{N_{t}\times 1}$ is additive white Gaussian noise (AWGN), $n∼\mathrm{CN}\left(0,\sigma^{2}\right)$, and $\sigma^{2}$ corresponds to noise power. Furthermore, combining Equations (1) and (2), we can further represent the received signals as

(3) $$r_{k}=h_{k}^{H}{\left(F_{\mathrm{RF}}F_{\mathrm{BB}}\right)}_{k}s_{k}+h_{k}^{H}\sum_{l\neq k}{\left(F_{\mathrm{RF}}F_{\mathrm{BB}}\right)}_{l}s_{l}+n.$$

**Figure 2:**
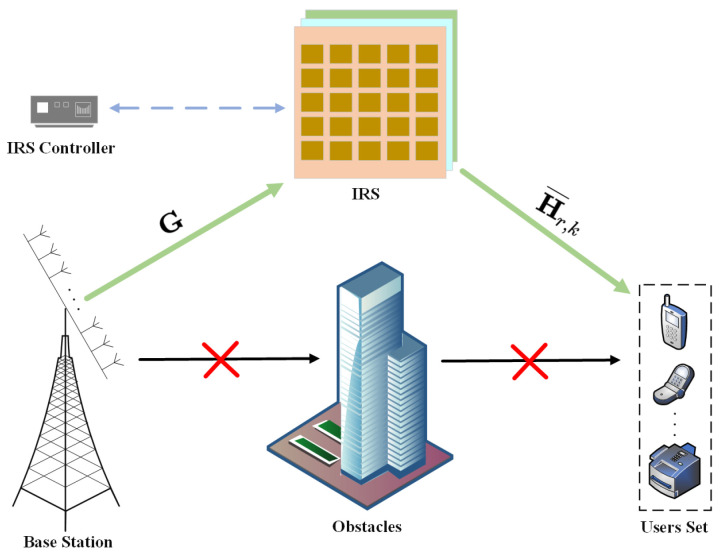
IRS-assisted multiple-users massive MIMO system.

With the increasing demand for quality of communication, to highlight the effectiveness of the proposed scheme in important and complex scenarios in the following simulation experiments, we utilize the popular IRS to establish a common communication system. In the practical application of IRS, it is found that IRS significantly improves the communication quality and sum rate in many complex applications such as an indoor scenario with some obstacles and a large number of users, an outdoor scenario where BSs are difficult to cover the users at the edge, a scenario where vehicle communication is in high-speed mobility, and a scenario where a large number of devices form a huge Internet of Things (IoT). Therefore, we simulate a multiple-users massive MIMO system with a certain obstacle, and thus, ignore the direct BS–users set link, focusing on the application scenario of the IRS communication link, which is highlighted by the brief composition of the established system in Figure 2.

In Figure 2, we can see the main hardware structure of the IRS, which consists of a three-layer composite panel and an IRS controller. The composite panel has the advantage of being corrosion-resistant, durable, and easy to penetrate, as well as having a low hardware cost. The first layer of the board is a dielectric matrix board with M×M reflective elements, meta atoms with specific shapes and sizes embedded in specific orientations, which can be digitally encoded to adjust the incident signals in real time. The second layer of the board is the control circuit board, which can intelligently adjust the amplitude/phase of each IRS element after receiving the command from the controller, thus achieving the powerful function of wireless signal multipath transmission. The third layer of the board is often made of brass or other metal materials, mainly to avoid signal energy overflow during IRS operation, resulting in reflective effect deviation. In reality, a field programmable gate array (FPGA) is often deployed as the IRS controller to intelligently control the IRS through the wireless signals while receiving or feeding back information to the massive MIMO BS as well as the users set at a relatively low sum rate, which means that the entire IRS-assisted system has an almost negligible operational overhead [24]. Since the research in this paper focuses on the later HBF scheme SOA-HBF, based on the subset optimization algorithm SOA, in order to simplify the system model we assume that there is no coupling effect between the IRS elements, the reflected signals received by the users set are the cumulative radiation effect of the elements, and at the same time, the positional parameters such as the deployment angle and distance of the IRS are not introduced in this paper [25].

Next, we need to simulate the channel model of the IRS-assisted system. The Rayleigh fading channel, as the most classical and basic flat fading channel, is still of great importance for research in B5G. The Rayleigh channel model has the following three main characteristics: Firstly, the Rayleigh channel is mainly used to simulate urban and indoor propagation environments containing a large number of buildings and other obstacles, and its signal power spectrum obeys the Rayleigh distribution in the frequency domain. Secondly, the transmission path in the Rayleigh channel contains a lot of scattering, bypassing, and reflections other than the direct paths at the transmitter and receiver, and the scattering phenomenon of these signals will cause multipath effects, and thus, lead to fast fading of the channel. Thirdly, it is difficult to obtain a direct mathematical model of the Rayleigh channel in order to simulate the random signal fading in multipath channels, so the complex Gaussian stochastic process is commonly used to model it in the simulation process, and the real and imaginary parts represent the amplitude and phase of the signal, respectively [14,26]. We can use the random complex Gaussian channel to simulate the Rayleigh fading channel.

Compared with the Rayleigh fading channel, the Rician fading channel is more favored by industry because it can more accurately reflect the real-world wireless communication environment. There are two main characteristics of the Rician channel model: Firstly, the Rician channel is essentially a special kind of Rayleigh channel, the special feature is that it adds an LoS propagation path in the signal propagation process which is full of scattering, and the signals received by the receiver obey the Rician distribution. Therefore, the Rician channel is commonly used in application scenarios where there is a significant LoS component, such as satellite communications and outdoor communications. Secondly, the parameter Rician factor is introduced. Since the Rician channel contains a multipath component consisting of an LoS part and an NLoS part with different powers, the Rician factor is defined as the ratio of the powers of the two signal components. The Rician factor can reflect the channel quality more accurately and measure the fading degree of the channel in the time, space, and frequency domains, which is important for improving the transmission quality and stability of the system [27,28]. The expression for the Rician factor κ is

(4) $$\kappa =\frac{p_{1}}{2p_{2}},$$

where the above LoS and NLoS belong to two different application scenarios in the specific signaling process: the power of the LoS component is $p_{1}$ and the power of the NLoS component is $p_{2}$. Combining the mathematical properties of the Rician channel and the Rayleigh channel, we can see that the Rician channel degenerates to the Rayleigh channel when $p_{1}=0$, i.e., κ=0. And the mathematical expression for the Rician channel matrix can be expressed as

(5) $$H_{\mathrm{Ric}}=\sqrt{\frac{\kappa }{1+\kappa }}{\tilde{H}}^{\mathrm{LoS}}+\sqrt{\frac{1}{1+\kappa }}{\tilde{H}}^{\mathrm{NLoS}}.$$

Synthesizing the advantages and disadvantages of the two channel models discussed earlier as well as the applicable scenarios, the Rician channel has stable performance and rich application scenarios, being an enhanced version of the Rayleigh channel, and the Rician channel can also be used to model mmWave channel [29]. The key is that the signal transmitted by the BS–IRS and IRS–users set link all contain a large amount of scattering, and at the same time there exists direct radiation from the BS to a specific IRS element, and then, to a specific user when necessary, so we model the BS–IRS link and IRS–users set link as a Rician channel, and experimentally determine the Rician factor κ=10. The simulated channel matrix $G\in C^{M^{2}\times N_{t}}$ and ${\bar{H}}_{r,k}\in C^{K\times M^{2}}$ can be obtained by Equation (5). ${\tilde{H}}^{\mathrm{LoS}}={\dot{H}}^{\mathrm{LoS}}\in C^{M^{2}\times N_{t}}$ denotes a direct signal between the BS and IRS, and ${\tilde{H}}^{\mathrm{NLoS}}={\dot{H}}^{\mathrm{NLoS}}\in C^{M^{2}\times N_{t}}$ denotes the scattered signals in the BS–IRS link. Similarly, ${\tilde{H}}^{\mathrm{LoS}}={\ddot{H}}^{\mathrm{LoS}}\in C^{K\times M^{2}}$ denotes a direct signal between the BS and a user of the users set, and ${\tilde{H}}^{\mathrm{NLoS}}={\ddot{H}}^{\mathrm{NLoS}}\in C^{K\times M^{2}}$ denotes the scattered signals in the BS–users set link which can correspond well to each users subsets. It is worth mentioning that the LoS and NLoS components used above are random complex matrices of corresponding dimensions, and the elements ξ within the matrices all obey a complex Gaussian distribution with mean zero and variance one, i.e., they satisfy $\xi ∼\mathrm{CN}\left(0,1\right)$.

Each element in the IRS phase shift matrix $\Theta \in C^{M^{2}\times M^{2}}$ corresponds to an element on the IRS, and by changing the phases of these elements, the IRS can realize precise control of the reflected signals, thus achieving the purposes of beamforming, signal focusing, and suppressing the interference, etc. [30]. This system selects the random-phase algorithm used by Q. Wu et al. in [21]. It is implemented by generating a random array ρ with the length of the number of IRS elements and mapping the elements therein between [0,2π). Then, diagonal matrices Θ with complex exponentials for the diagonal elements are created, and the real part of these complex numbers is 1 and the imaginary part is the random phase at the corresponding position in ρ. This algorithm maps the random phases corresponding to the number of IRS elements onto the complex plane, thus creating a complex matrix that is a better fit to the Rician channel used for the IRS–users set link.

After determining the IRS phase-shift matrix as well as the BS–IRS link and the IRS–users set link, we can denote the channel matrix of the IRS-assisted system $H_{\mathrm{IRS}}\in C^{N_{t}\times K}$ as follows:

(6) $$H_{\mathrm{IRS}}=G^{H}\Theta {\bar{H}}_{r,k},$$

where the IRS phase-shift matrix $\Theta \in C^{M^{2}\times M^{2}}$. Finally, we declare that for convenience, in the subsequent description of the proposed scheme, as the two systems are not distinguished, we use $H\in C^{N_{t}\times K}$ to uniformly represent the two systems, including the direct BS–users set link and the cascaded channel of the IRS-assisted system. We define $H\in C^{N_{t}\times K}$ as the above two established systems’ channel matrix.

### 2.2. Problem Formulation

For the convenience of problem formulation, we use a famous linear precoding algorithm to obtain the digital beamformer. Considering that teh digital beamformer preprocessing of baseband signals is an important factor to improve the system’s sum rate and ensure the quality of signal transmission, we use a promising linear precoding algorithm RZF as the criterion for designing the digital beamformer $F_{\mathrm{BB}}$, respectively [13]. In Equation (3), the first term of the expansion is the signal that the receiver expects to receive, while the second term represents the multi-user interference (MUI), and the third term is the noise term. The receiver is not expecting to receive the second and third terms, which will affect the signal transmission quality. The goal of the linear precoding algorithm is to minimize the MUI as well as the AWGN to maximize the first term of the desired received signal for a given power. Note that in this paper, perfect CSI is assumed to be perfectly known at the BS.

The regularized zero-forcing algorithm combines the advantages of the ZF algorithm and the MMSE algorithm and mitigates the effects of their shortcomings, so the RZF algorithm has a strong attraction for researchers and is also widely deployed in real-world application scenarios. The ZF algorithm is one of the most classical precoding algorithms, which can achieve excellent system performance with low computational complexity, but ignores the problem of noise. Thus, while ZF eliminates the inter-user interference, the energy of the received signal is expected to be reduced at the receiver side, thus leading to the amplification of the noise. The MMSE algorithm complements the ZF algorithm and is no less important than the ZF algorithm. Unlike the ZF algorithm, which is forced to zero out the noise amplification problem caused by interference, the MMSE algorithm effectively mitigates this problem. The MMSE algorithm integrates the effects of noise variance and inter-user interference and optimizes the transmission performance of the system by minimizing the mean square error of the received signals. Even in scenarios with poor SNR, this solution can bring considerable gains to the system, but it brings high computational complexity that is difficult to ignore. The RZF algorithm introduces a variable regularization parameter ϱ on the basis of the ZF algorithm. The digital beamformer $F_{\mathrm{BB}}$ is obtained according to the RZF precoding algorithm as

(7) $$F_{\mathrm{BB}}=\hat{H}{\left({\hat{H}}^{H}\hat{H}+\varrho I_{K}\right)}^{-1},$$

where $\hat{H}=F_{\mathrm{RF}}^{H}H\in C^{N_{\mathrm{RF}}\times K}$ and the value of ϱ ranges from zero to one. The RZF algorithm degrades to the ZF algorithm when the regularization parameter ϱ=0, and the RZF algorithm degrades to the MMSE algorithm when the regularization parameter ϱ=1. As an optimization parameter that weighs the performance and computational complexity of the precoder, the determination of this regularization parameter should often take into account a variety of factors, such as the deployment scale, the application environment, the user’s needs, the hardware facilities, and it needs to be accompanied by a large number of experiments to verify it [31,32,33].

In conjunction with the precoding algorithm we discussed earlier, $F_{\mathrm{BB}}$ can be obtained by associating H with $F_{\mathrm{RF}}$, which allows the HBF matrix design to be transformed into the ABF matrix design. In this part, we present the optimization problem aiming to obtain the optimized analog beamformer $F_{\mathrm{RF}}^{*}$ under certain constraints and with the objective of the system’s sum-rate maximization [34]. The system’s sum rate can be expressed as

(8) $$R=\sum_{k=1}^{K}\log_{2}\left(1+\Psi {\left(F_{\mathrm{RF}},F_{\mathrm{BB}}\right)}_{k}\right).$$

where $\Psi {\left(F_{\mathrm{RF}},F_{\mathrm{BB}}\right)}_{k}$ corresponds to the signal-to-interference-noise ratio (SINR) of the user *k*, and SINR can be calculated as

(9) $$\Psi \left({\left(F_{\mathrm{RF}},F_{\mathrm{BB}}\right)}_{k}\right)=\frac{{\left|h_{k}^{H}{\left(F_{\mathrm{RF}}F_{\mathrm{BB}}\right)}_{k}\right|}^{2}}{\sum_{l\neq k}{\left|h_{k}^{H}{\left(F_{\mathrm{RF}}F_{\mathrm{BB}}\right)}_{k}\right|}^{2}+\sigma^{2}}.$$

Then, we use the commonly used DFT codebook F to represent a set of feasible ABF solutions [35], which can be expressed as

(10) $$\begin{array}{cc} F=\{a(\varphi_{i})| & [\sin\varphi_{1},\sin\varphi_{2},\cdots ,\sin\varphi_{N_{t}}]=\\ & [0,-\frac{2}{N_{t}},\cdots ,-\frac{N_{t}}{N_{t}},-\frac{N_{t}+2}{N_{t}}+2,\cdots ,-\frac{2(N_{t}-1)}{N_{t}}+2]\}. \end{array}$$

where the computation of the ULA antenna array response vectors $a_{t}\left(\varphi \right)$ can be expressed as

(11) $$a_{t}\left(\varphi_{i}\right)=\sqrt{\frac{1}{N_{t}}}{\left[1,e^{-j2\pi \frac{d}{\lambda }\sin\varphi^{i}},\ldots ,e^{-j2\pi \frac{d}{\lambda }\left(N_{t}-1\right)\sin\varphi^{i}}\right]}^{T},$$

where λ is the carrier wavelength and the distance between neighboring antenna elements in the ULA at BS is equal to half of the carrier wavelength, i.e., d=0.5λ.

In the codebook F, if a coordinate system is established for a massive MIMO BS perpendicular to the ground and BS is the *X*-axis, the codebook can cover $\left[0,180^{\circ }\right]$ in the direction of the antenna transmitting signals (corresponding to $\sin\varphi_{i}\in \left[-1,1\right]$), with a minimum signal interval of $\sin\frac{2}{N_{t}}$. This provides a sufficient set of beamforming vectors for us to find the optimized analog beamformer, so the corresponding optimization problem in this paper can be written as

(12) $$\begin{array}{c} \begin{array}{cc} \max_{F_{\mathrm{RF}}^{*}}\; & \sum_{k=1}^{K}\log_{2}\left(1+\frac{{\left|h_{k}^{H}{\left(F_{\mathrm{RF}}F_{\mathrm{BB}}\right)}_{k}\right|}^{2}}{\sum_{l\neq k}{\left|h_{k}^{H}{\left(F_{\mathrm{RF}}F_{\mathrm{BB}}\right)}_{k}\right|}^{2}+\sigma^{2}}\right),\\ s.t.\; & {\left(F_{\mathrm{RF}}\right)}_{k}\in F. \end{array} \end{array}$$

The optimization problem Equation (12) is a convex optimization problem, and we use the proposed SOA algorithm-based HBF scheme SOA-HBF in the next section to solve it.

## 3. An HBF Scheme Based on Subset Optimization Algorithm

In this section, inspired by the algorithms proposed in [36,37], we develop an HBF scheme based on the proposed subset optimization algorithm SOA, which can effectively reduce the inter-user interference, and thus, improve the system’s sum rate. The implementation of the SOA algorithm consists of two stages: In the first stage, according to the channel correlation, the users are separated into different numbers of users subsets with similar correlations. In the second stage, the optimization problem Equation (12) is combined to traverse the beamforming vectors in the codebook in terms of different subsets. An exhaustive traversal of the set of vectors in terms of users in the second stage of SOA may lead to extremely high computational complexity, and it is foreseeable that a different codebook will greatly affect the performance of the system when using this algorithm. Therefore, in our proposed algorithm the selected vectors are removed from the codebook after the selection is performed sequentially on a user subset basis. Although it still generates some computational complexity, considering that massive MIMO is characterized by a much lower number of users than antennas, each subset of users separated from the users set will not be too large and the number of users in the subset will be small, so the computational complexity brought about by it is tolerable, and at the same time, this method can partially eliminate the impact of the codebook on the performance of the system.

### 3.1. First Stage of SOA

We know that the existence of inter-user interference will have a large impact on the transmission performance of the system; in the case of large interference, the users should work together to select the uniform beamforming vector to avoid exacerbating the interference, and thus, further affecting the system performance. Inter-user correlation indicates the degree of correlations between users, and interference is positively correlated with inter-user correlation. When the correlation between two users is high, they will have similar transmission characteristics and signal waveforms, which is the reason for the existence of inter-user interference. Therefore, we believe that dividing different users subsets in the users set by correlation and collaboratively selecting the analog beamforming vectors with the largest system’s sum rate in the codebook F in terms of the subsets is very reliable for effectively controlling and minimizing user interference. It is worth mentioning that the spatial correlations between users is also a factor affecting the interference, and the spatial distance between users is inversely correlated with the interference, but the spatial distance and other parameters are not the focus of this paper, and we assume here that the *K* users in the users set keep equal spatial distances from each other, and the assumptions are kept unchanged after the set is divided into subsets.

The inter-user correlation can be calculated by the channel correlation; we use a matrix $\Xi \in R^{K\times K}$ to correspond one by one the correlations between the user $\dot{k}$$\left(\dot{k}=1,2,\cdots ,K\right)$ and the user $\dot{k}$, $\left(\ddot{k}=1,2,\cdots ,K\right)$, and the elements of the diagonal of the matrix, i.e., the correlations between the same users, shall be one; where the correlations matrix can be written as

(13) $$\Xi_{\dot{k},\ddot{k}}=\left\{\begin{array}{cc} {\left|H_{\dot{k}}^{H}H_{\ddot{k}}\right|}^{2} & \dot{k}\neq \ddot{k},\\ 1 & \dot{k}=\ddot{k}. \end{array}\right.$$

The property of the matrix determines that there will be $\Xi_{\dot{k},\ddot{k}}=\Xi_{\ddot{k},\dot{k}}$, and in order to avoid errors in subsequent calculations arising from two perfectly equal inter-user correlations, we will retain the value of the lower triangular element of the Ξ matrix and make the rest of the elements zero, resulting in a new matrix of $\Xi^{D}$. Next, the user pair $k_{1}$ and $k_{2}$, corresponding to the maximum value found using the correlations matrix, can be written as

(14) $$\left(k_{1},k_{2}\right)=arg\max_{\dot{k},\ddot{k}}{\left(\Xi^{D}\right)}_{\dot{k},\ddot{k}}.$$

We use $\hat{U}$ to refer to the users subsets, and divide the above two users within the subset, i.e., $\hat{U}=\left\{k_{1},k_{2}\right\}$, assuming that the number of users within a particular subset after completing the subset division once is greater than two, i.e., $\hat{U}=\left\{k_{1},k_{2},\cdots ,k_{i}\right\}$; here it is i∈Z. The following is the detailed process of deciding to divide $k_{i}$ into a subset after dividing users $k_{1},k_{2}$ into the subset. We consider a parameter T as a threshold for whether or not to divide a user into the candidate set; $T_{i}$ is obtained by taking the average of the correlations of user $k_{i}$ and the rest of the K−1 users, which can be expressed as

(15) $$T_{i}=\frac{\sum_{k_{\hat{i}}=1}^{K}\Xi_{k_{i},k_{\hat{i}}}^{D}}{K}.$$

Using the threshold T, we further divide the remaining K−i users in the users set *U* by filtering the users $k_{\hat{i}}$ whose correlations with user $k_{i}$ are greater than the threshold $T_{i}$ and placing them into the candidate set $\Delta_{i}$ first, which can be written as

(16) $$\Delta_{i}=\left\{k_{\hat{i}}|\Xi_{k_{i},k_{\hat{i}}}^{D}>T_{i},k_{\hat{i}}\in U\right\}.$$

In order to make the correlation between the newly added user and all the users in the subset $\hat{U}$ greater than the corresponding threshold, the total set of candidates is the intersection of the candidate subsets, i.e., $\Gamma =\left\{\Delta_{1}\cap \Delta_{2}\cap \cdots \Delta_{i}\right\}$. Then, we decide whether to divide the i+1th user from *U* into $\hat{U}$ based on the number of elements in the total set of candidates Γ. If Ω(Γ)=0, it means that the subset has been divided and contains *i* users. If $\Omega (\Gamma_{i})=1$, the users in Γ can be directly divided into the subset, which then contains i+1 users. If Ω(Γ)>1, we can assume that the candidate set contains *i* elements, i.e., $\Gamma =\left\{{\bar{k}}_{1},{\bar{k}}_{2},\cdots ,{\bar{k}}_{i}\right\}$. We then calculate the sum of correlations between user $\bar{k}$ and user *k* in the subset using the following equation:

(17) $$\Upsilon_{k,\bar{k}}=\sum_{i=1}^{i}\Xi_{k_{i},\bar{k}}^{D},\bar{k}\in \Gamma .$$

Then, find the index at $\Upsilon_{k,\bar{k}}$ that corresponds to the maximum value as the i+1th user of the subset:

(18) $$k_{i+1}=\arg\max_{\bar{k}}\Upsilon_{k,\bar{k}}.$$

The two cases in Ω(Γ)≠0 indicate that this subset division is not finished, and we need to consider the i+2th user of the subset. First, a new candidate set $\Delta_{i+1}$ is obtained using $T_{i+1}$ computed by Equation (15), and then, the total set of candidates $\Delta_{i+1}$ of the i+2th user of the subset is updated by $\Gamma =\left\{\Gamma \cap \Delta_{i+1}\right\}$. Finally, we repeat the above steps until Ω(Γ)=0 to end this subset division.

Since the main purpose of dividing subsets in the first stage is to eliminate inter-user interference, we effectively initialize the correlations matrix $\Xi^{D}$ after each completed subset division. Taking a subset ${\hat{U}}_{1}=\left\{k_{1},k_{2}\right\}$ that contains two users and has been divided as an example, we set all the elements in row one, row two, column one, and column two of order $\Xi^{D}$ to be zero. In addition to this, before the next subset division starts formally, intermediate sets such as the total set of candidates Γ should be reduced to the empty set. Finally, the above steps are repeated until $\Xi^{D}=0$, which means that all the users have been fully divided and can start the second stage of SOA for beamforming vector selection.

### 3.2. Second Stage of SOA

After the previous stage of user division, the original set containing *K* users has been optimized into *u* subsets. Next, we perform simulated beamforming vector selection in the codebook F in each subset. We assume in turn that subset ${\hat{U}}_{1}$ contains $U_{1}$ users, subset ${\hat{U}}_{2}$ contains $U_{2}$ users ⋯ and subset ${\hat{U}}_{u}$ contains $U_{u}$ users; it can be shown that $U_{1}+U_{2}+\cdots +U_{u}=K$. Starting from subset ${\hat{U}}_{1}$, considering the requirement of maximizing the system’s sum rate in the optimization problem Equation (12) we first compute the beamforming gains *w* to be used as the basis for selecting analog beamforming vectors from F, which can be written as

(19) $$w_{u}=\frac{\left|{h_{u}}^{H}Wh_{u}\right|}{N_{t}^{2}},$$

where $W\in C^{N_{t}\times N_{t}}$ represents a beamforming matrix obtained from the codebook F. The expression $\frac{h_{u}}{N_{t}}$ denotes the average channel gains within the users subset. Subsequently, employing a stable algorithm merge sort, the vectors from the corresponding codebook are sorted in ascending order based on the magnitude of *w*. The subsequent step involves selecting the last $U_{1}$ beamforming vectors and placing them into the subcodebook $F_{1}$. Finally, by utilizing indices, a dedicated beamforming matrix ${\tilde{W}}_{1}\in C^{N_{t}\times N_{t}}$ is constructed from W, extracting individual rows and columns as pertaining to the subset ${\hat{U}}_{1}$. Except for the newly introduced elements, the remaining elements in ${\tilde{W}}_{1}$ are set to zero. Simultaneously, the elements at corresponding positions in W are set to zero, reducing the computational complexity for the subsequent subset’s selection of beamforming vectors from the codebook. The time and space complexities of the merge sort employed here are both $O(U_{1}\log U_{1})$.

Next, we compute $\left|{h_{j}}^{H}{\tilde{W}}_{1}h_{j}\right|$ within the subset by using the corresponding user channel and beamforming matrix, with the corresponding $j=1,2,\cdots ,U_{1}$. The computed gains coefficients are used to match the users in the subset with the vectors in $F_{1}$ based on the principle of maximizing the system’s sum rate, and the computational complexity of this step is $O(U_{1}^{2})$. As the traversal of the subset ${\hat{U}}_{1}$ is completed to obtain the corresponding optimized analog beamforming vector $w_{1}\in C^{N_{t}\times U_{1}}$, one can start repeating the above steps for the following u−1 subsets.

Finally, after all subsets have completed vector selection, we obtain the optimized analog beamformer required for the optimization problem Equation (12):

(20) $$F_{\mathrm{RF}}^{*}=\left[w_{1},w_{2},\cdots ,w_{u}\right],$$

where $w_{u}=\left[a\left(\varphi_{1}\right),a\left(\varphi_{2}\right),\cdots ,a\left(\varphi_{U_{u}}\right)\right]$, and $a\left(\varphi \right)\in C^{N_{t}\times 1},a\left(\varphi_{U_{u}}\right)\in F_{u}$.

### 3.3. SOA-Based HBF Scheme SOA-HBF

In conjunction with the above subset optimization-based algorithm SOA (Algorithms 1 and 2), we first give here the complete runtime pseudo-code corresponding to the two stages of user division and vector selection.

And after the SOA algorithm has been run entirely, according to the RZF precoding algorithm, we can use Equations (7) and (20) to calculate the optimized digital beamformer $F_{\mathrm{BB}}^{*}$. Furthermore, $\hat{H}=F_{\mathrm{RF}}^{H}\tilde{H}\in C^{K\times K}$, while for $\tilde{H}\in C^{N_{t}\times K}$ compared to the channel matrix H the only difference is the order of the elements, which we obtained based on the new user correspondences in the optimized users subset. In addition, we set the regularization factor ϱ to 0.05, which was determined after experimental verification and is presented in the simulation results of the next section.

**Algorithm 1** First Stage of SOA Algorithm
**Require:** $h_{\dot{k}}$, $h_{\ddot{k}}$ and *K*, $\left(\dot{k},\ddot{k}=1,2,\cdots ,K\right)$.**Ensure:** User Subsets (${\hat{U}}_{1},{\hat{U}}_{2},\cdots ,{\hat{U}}_{u}$).1:Calculate Equation (13) for Ξ;2:Preserve the elements of down triangular matrix for $\Xi^{D}$;3:
**repeat**
4:    Initialize intermediate sets Γ and Δ;5:    Calculate Equation (14) to divide *i* users in $\hat{U}$;6:    Calculate Equation (15) for T and utilize Equation (16) to determine which users from remaining K−i users can be divided in candidate subsets Δ;7:    Update candidate sets by $\Gamma =\left\{\Delta_{1}\cap \Delta_{2}\cap \cdots \Delta_{i}\right\}$;8:    **if** Ω(Γ)>1 **then**9:        Calculate Equation (17) for Υ and utilize Equation (18) to find the (i+1)-th user in $\hat{U}$;10:    **else if** Ω(Γ)=1 **then**11:        Directly divide this user in $\hat{U}$;12:    **else**13:        **break**;14:    **end if**15:    **while** Ω(Γ)≠0 **do**16:        Go to Step 6;17:    **end while**18:**until** $\Xi^{D}=0$19:**return** ${\hat{U}}_{1},{\hat{U}}_{2},\cdots ,{\hat{U}}_{u}$.


**Algorithm 2** Second Stage of SOA Algorithm
**Require:** $N_{t}$, W from F, (${\hat{U}}_{1},{\hat{U}}_{2},\cdots ,{\hat{U}}_{u}$) and corresponding $\left(h_{1},h_{2},\cdots ,h_{u}\right)$.**Ensure:** Optimized Analog Beamformer $F_{\mathrm{RF}}^{*}$.1:**for**i=1,2,...,u **do**2:    Calculate Equation (19) for *w*;3:    Using *w* to sort corresponding beamforming vectors in F by Merge-sort;4:    Select and place the last $U_{i}$ beamforming vectors into the subcodebook $F_{i}$;5:    Place all elements of the corresponding row and column in W into the same position in a all zero matrix ${\tilde{W}}_{i}$ of the same dimension;6:    Set the corresponding row and column elements of W to 0;7:    **for** $j=1,2,...,U_{i}$ **do**8:        Calculate $\left|{h_{j}}^{H}{\tilde{W}}_{i}h_{j}\right|$ to match users in ${\hat{U}}_{i}$ with beamforming vectors in $F_{i}$ according to the principle of maximizing system capacity;9:    **end for**10:
**end for**
11:**return** $\left[w_{1},w_{2},\cdots ,w_{u}\right]=F_{\mathrm{RF}}^{*}$.


The optimized hybrid beamformer $F^{*}$ obtained by our proposed SOA-based HBF scheme SOA-HBF satisfies the system’s sum-rate maximization requirements

(21) $$F^{*}=F_{\mathrm{RF}}^{*}F_{\mathrm{BB}}^{*}=arg\max_{F_{\mathrm{RF}}^{*},F_{\mathrm{BB}}^{*}}\sum_{k=1}^{K}\log_{2}\left(1+\Psi {\left(F_{\mathrm{RF}}^{*},F_{\mathrm{BB}}^{*}\right)}_{k}\right).$$

After deploying the optimized beamformer $F^{*}$ obtained from the proposed SOA-HBF scheme in the BS, it improves the performance and reliability of the wireless communication system by optimizing the directionality, anti-interference, and multipath suppression of the transmitted signal. This is crucial for improving the capacity and quality of communication systems and we prove this viewpoint in the following simulation experiments.

## 4. Simulation Results

In this section, we first give the simulation results related to the regularization factor of the RZF precoding algorithm determined in the previously proposed HBF scheme. Then, the performance of the proposed HBF scheme based on the subset optimization algorithm SOA is verified by simulation experiments from four perspectives of SNR: number of users, number of BS antennas, and number of IRS elements in terms of system’s sum rate in the established IRS-assisted system. Finally, to verify the adaptability of the proposed scheme, we deploy the proposed scheme in a direct-connected mmWave communication system without obstacles, and carry out same the simulation experiments from three perspectives of SNR: number of users, number of BS antennas, and in terms of system’s sum rate. In addition, we also use classical HBF schemes including the OMP scheme [38], MO scheme [39], Greedy scheme [40] and finite-resolution scheme [41] as baselines under the same configuration of the system model in order to highlight the superior performance of the proposed scheme. It is worth mentioning that the experiment also introduced a full-digital beamforming scheme as the upper bound of the system’s sum rate.

In the default settings of the system model, $N_{t}=64,N_{s}=N_{\mathrm{RF}}=K=8,$ SNR = 20 dB, $N_{cl}=5,N_{ray}=10,\kappa =10,\varrho =0.05$, and the IRS is embedded with 81 uniformly arranged elements of 9×9. As for the subsequent mmWave channel model, except for the settings mentioned above, they are consistent with the simulation experiment settings in [42]. All simulation results in this section were obtained through simulation experiments using MATLAB R2022b.

### 4.1. Determination of RZF Regularization Factors

Considering the range of values of the RZF regularization factor, we experimentally verified the default setting in the HBF scheme SOA-HBF based on the SOA algorithm by replacing the value of ϱ. The following results show the representative values of ϱ in different ranges for comparison. The horizontal axis is taken as an SNR of [−20 dB,20 dB] with a step size of 5 and the vertical axis is the sum rate in bps/Hz.

**Figure 3:**
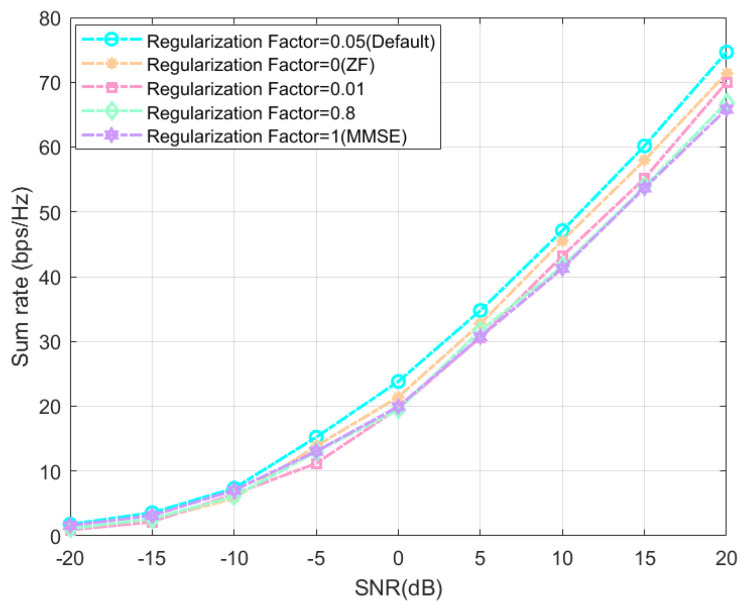
System’s sum rate of different RZF regularization factors at different SNR.

In Figure 3, we can intuitively feel that the proposed scheme consistently outperforms the others with different regularization factors selected under the default settings. It is worth mentioning that the simulation results fit well with the characteristics of the MMSE and ZF precoding algorithms, where the sum rate using MMSE precoding outperforms that of ZF precoding when the SNR is in a small range, while the ZF precoding approach shows a large performance improvement when the SNR is in a large range. This corresponds to the intersection of the ZF curve with the MMSE curve produced by the SNR around −5 dB in the figure. We can also conclude that the RZF precoding algorithm is closer to the MMSE precoding algorithm when the value of ϱ is closer to 1, and the RZF precoding algorithm is closer to the ZF precoding algorithm when the value of ϱ is closer to 0. We have found a balance point for the system model, i.e., when ϱ=0.05, the RZF precoding takes into account the reduction of the inter-user interference as well as noise with lower computational complexity, and has a good performance in different SNR ranges.

In addition, we can find that the difference between the ZF and MMSE precoding algorithms is not significant in the low SNR range. Usually, in the low SNR range, if the users set range is small, it results in small user spacing, or the equipment size limitation makes the different receiving antennas of the same user at the receiving end not have enough distance from each other, which will cause the receiving antennas to generate very strong channel correlations with each other and an ill-conditioned channel matrix will appear. Then, the ZF inverse will have an intolerable noise amplification problem that seriously affects the performance of the ZF precoding, whereas the noise amplification is not so bad because we set the users as a single receiving antenna [43].

### 4.2. The Impact of Four Perspectives on IRS-Assisted System’s Sum Rate

**Figure 4:**
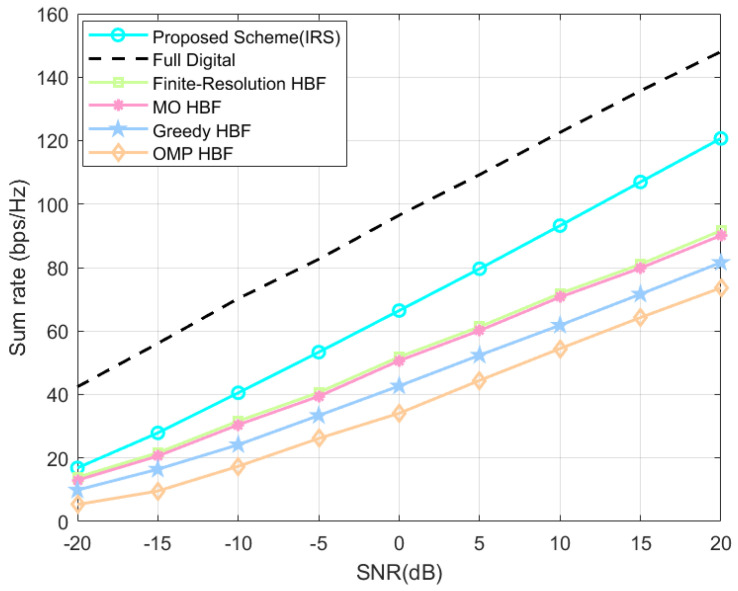
IRS-assisted system’s sum rate at different SNRs.

Shown in Figure 4 are the system’s sum-rate variation plots of the proposed HBF scheme based on the SOA algorithm used in an IRS-assisted massive MIMO system with different SNRs, and the other baseline schemes are also replaced with the same system model settings to highlight the advantages of the algorithms. The proposed scheme consistently outperforms the baseline HBF schemes in all SNR ranges. At the system default setting, i.e., SNR = 20 dB, the proposed scheme achieves system’s sum-rate improvements of about 24.1%, 25.3%, 32.4%, and 39.1% compared to the finite-resolution scheme, the MO scheme, the Greedy scheme, and the OMP scheme.

Interestingly, we can see that the classical numerical schemes such as the OMP scheme deployed in the IRS-assisted system model established in this paper have system gains of 18.46 bps/Hz after IRS is added to the system model, but the proposed scheme is as high as 46.04 bps/Hz; we cannot think that this is all due to the difference in the methodology, and it is partly because of the fact that it is difficult to emphasize all the advantages of traditional numerical scheme under the new technology, while our proposed scheme undoubtedly has good adaptive characteristics and can bring good system gains in new application scenarios.

**Figure 5:**
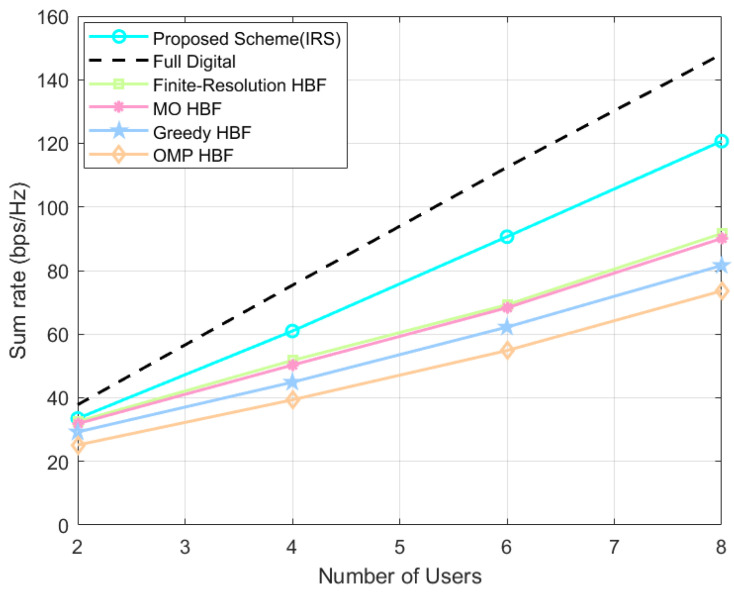
IRS-assisted system’s sum rate at different number of users.

As shown in Figure 5, the IRS-assisted system’s sum-rate variation curves are plotted when the number of users $K\in \left\{2,4,6,8\right\}$, the proposed scheme always outperforms the baseline HBF schemes with the same system settings. As the number of users increase, the system’s sum rate of the proposed scheme improves about 0.99–29.05 bps/Hz, 1.63–30.59 bps/Hz, 4.25–39.10 bps/Hz, 8.35–47.09 bps/Hz (all retained to two decimal places) compared to those of the finite-resolution scheme, the MO scheme, the Greedy scheme, and the OMP scheme. From the above differences, it can be seen that as the number of users increases, the gap between the proposed scheme and other HBF schemes becomes larger, so it can be predicted that, without loss of generality, the more the number of users, the more significant is the performance gain brought by the proposed scheme.

**Figure 6:**
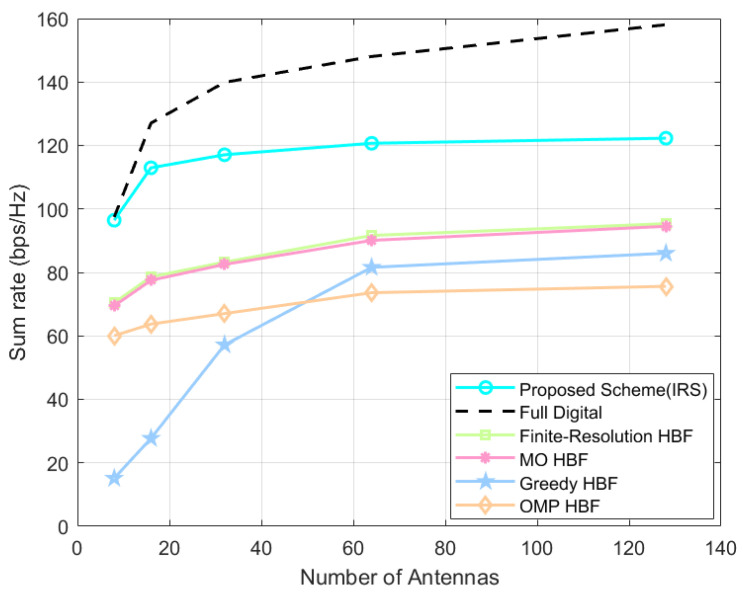
IRS-assisted system’s sum rate at different number of antennas.

As shown in Figure 6, which is a graph of the IRS-assisted system’s sum-rate variation when the number of BS antennas $N_{t}\in \left\{8,16,32,64,128\right\}$, the proposed scheme is always superior to the baseline HBF scheme with the same system settings. Here, although the proposed scheme does not increase the sum rate as much as the other schemes, especially the Greedy scheme, when the number of antennas is increased, the amplitude of gains is kept small when the number of antennas is increased from 64 to 128 at the end, and if the number of antennas is increased further, it cannot exceed the system’s sum rate of the proposed scheme.

**Figure 7:**
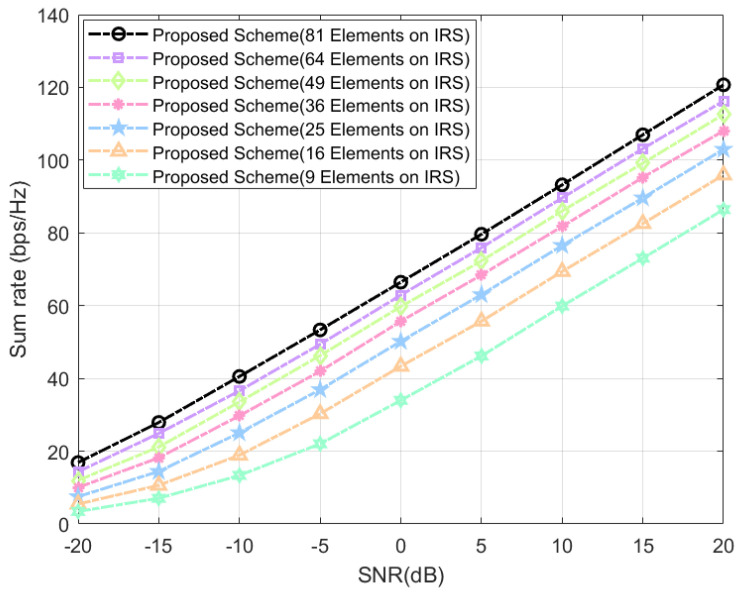
IRS-assisted system’s sum rate at different number of IRS-elements.

As shown in Figure 7, we observe the system gain by varying the number of elements on the IRS. The curve corresponding to 81 elements, which is our default setting, outperforms the other six curves with different element numbers. Intuitively, as the number of elements increases, the system’s sum rate improves; however, the rate of improvement diminishes. This trend is expected because our simulations do not take into account the mutual coupling effect between the IRS elements, i.e., the interference between the elements is ignored. In practice, mutual coupling is unavoidable and affects the results. Therefore, it is meaningful for IRS to consider the factor of mutual coupling when optimizing deployment plans in practical applications.

### 4.3. The Impact of Three Perspectives on Traditional Direct Link System’s Sum Rate

After completing the SOA-HBF-based simulation experiments on the established IRS-assisted massive MIMO system with obstacles. The mmWave channel, operating between 30 GHz and 300 GHz, offers abundant spectrum resources for increased user access. Its high bandwidth ensures a fast transmission rate and low delay, making it vital for B5G. Using a continuous and smaller beam reduces obstacles, interference, and bit error rate, enhancing communication efficiency. However, its limited transmission distance and susceptibility to environmental factors make it suitable for specific scenarios like high-speed vehicle communication and densely populated urban areas [44]. We use a popular mmWave channel model to represent direct connected systems when there are no obstacles between communication systems, and continue to use the proposed SOA-HBF in the following experiments.

**Figure 8:**
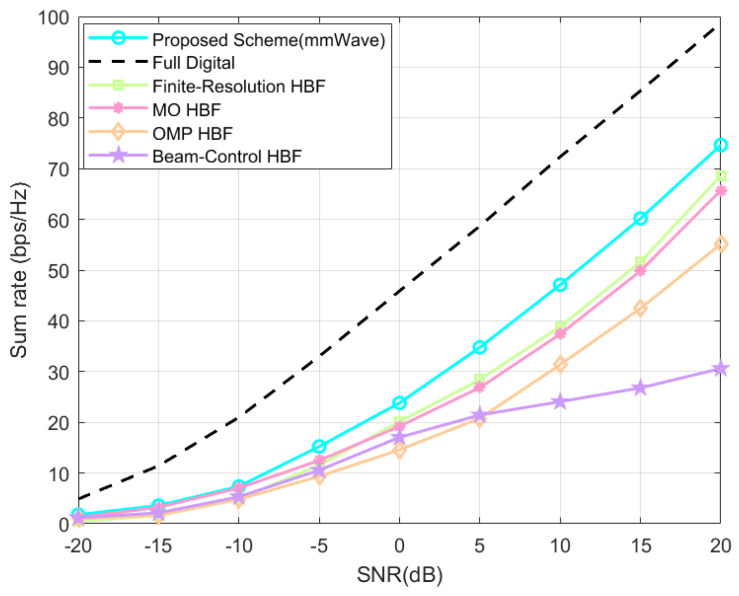
Direct link system’s sum rate at different SNR.

Shown in Figure 8 are the system’s sum-rate variation plots of our proposed HBF scheme based on the SOA algorithm are used in a massive MIMO system with direct link in the mmWave channel with different SNRs. The proposed scheme consistently outperforms the baseline HBF schemes in all SNR ranges. At the default settings of the system, i.e., SNR = 20 dB, the system’s sum rates of the proposed scheme are improved by about 8.1%, 11.9%, 26.1%, and 59.1% (all retained to one decimal place) compared with the finite-resolution scheme, the MO scheme, the OMP scheme, and the beam-control scheme.

**Figure 9:**
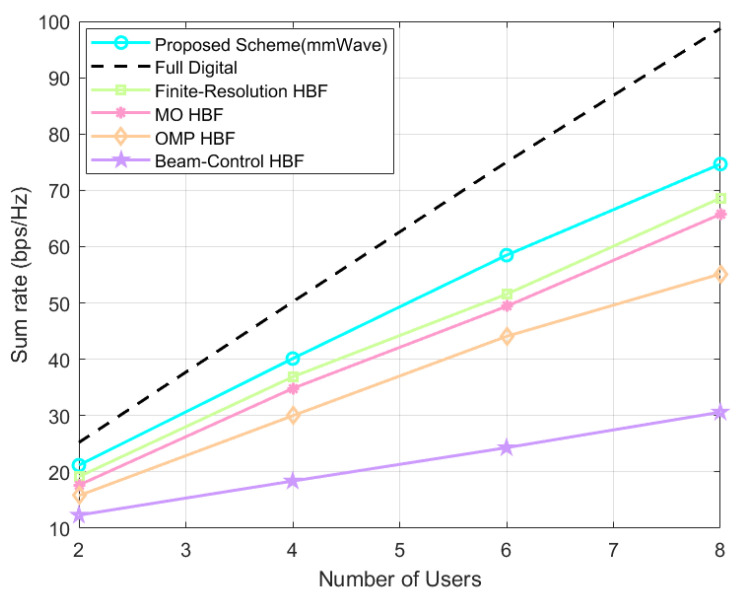
Direct link system’s sum rate at different number of users.

As shown in Figure 9, which is a graph of the system’s sum-rate variation when the number of users $K\in \left\{2,4,6,8\right\}$, the proposed scheme always outperforms the baseline HBF schemes. As the number of users increases, the system’s sum rate of the proposed scheme improves by about 2.03–6.04 bps/Hz, 3.53–8.92 bps/Hz, 5.41–19.50 bps/Hz, 8.87–44.04 bps/Hz (all retained to two decimal places) as compared to that of the finite-resolution scheme, the MO scheme, the OMP scheme, and the beam-control scheme. From the above differences, it can be seen that as the number of users increases, the gap between the proposed scheme and other HBF schemes becomes larger, so it can be predicted that, without loss of generality, the more the number of users, the more significant the performance gains brought by the proposed scheme.

**Figure 10:**
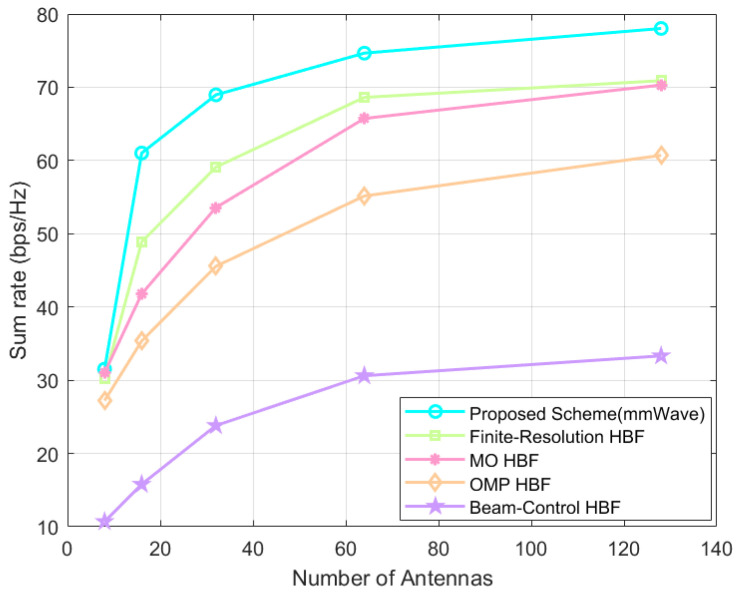
Direct link system’s sum rate at different number of antennas.

As shown in Figure 10, when the number of BS antennas $N_{t}\in \left\{8,16,32,64,128\right\}$, the proposed scheme always outperforms the baseline HBF schemes. It should be noted that both our proposed HBF scheme based on the SOA algorithm and the baseline schemes bring much smaller sum-rate gains compared to the previous one when the transmitting antennas grow from 64 to 128, which is due to the inter-antenna interference and the increase in power consumption.

In Figure 10, which is a graph of the IRS-assisted system’s sum-rate variation when the number of BS antennas $N_{t}\in \left\{8,16,32,64,128\right\}$, the proposed scheme is always superior to the baseline HBF scheme with the same system settings. Here, although the proposed scheme does not increase the sum rate as much as the other schemes, especially the Greedy scheme, when the number of antennas is increased, the amplitude of gains is kept small when the number of antennas is increased from 64 to 128 at the end, and if the number of antennas is increased further, it cannot exceed the system’s sum rate of the proposed scheme.

## 5. Conclusions

In this paper, we proposed a hybrid beamforming scheme SOA-HBF based on the subset optimization algorithm SOA, which reduced the inter-user interference by dividing the users set into a certain number of users subsets to obtain certain gains in the system’s sum rate. By establishing an IRS-assisted massive MIMO system with obstacles under a fully connected hybrid beamforming architecture, which is a commonly used scenario for real-world applications, and verifying the proposed scheme and its superior performance and adaptability in different scenarios through simulation experiments from various perspectives, it is believed that it can provide a valuable solution for the development of hybrid beamforming technology.

## Data Availability

Data are contained within the article.
